# NINJ1 and MMP9: potential biomarkers for intracranial atherosclerosis plaque vulnerability

**DOI:** 10.3389/fneur.2025.1552948

**Published:** 2025-04-28

**Authors:** Xiao-lian Wei, Xin Da, Yu-ge Zhang, Zi-ang Li, Bing-jie Liu, Rui-fang Yan, Hua Zhong, Bin Yuan

**Affiliations:** ^1^Department of Neurology II, The First Affiliated Hospital of Xinxiang Medical University, Xinxiang, China; ^2^Henan Key Laboratory of Neural Regeneration, The First Affiliated Hospital of Xinxiang Medical University, Xinxiang, China; ^3^Department of Radiology Center, The First Affiliated Hospital of Xinxiang Medical University, Xinxiang, China; ^4^Department of Clinical Laboratory, The First Affiliated Hospital of Xinxiang Medical University, Xinxiang, China

**Keywords:** nerve injury-induced protein 1, matrix metalloproteinase 9, high-resolution vessel wall imaging, symptomatic intracranial atherosclerotic stenosis, plaque

## Abstract

**Background and objective:**

To utilize high-resolution vessel wall imaging (HR-VWI) to identify the characteristic features of culprit plaques in intracranial atherosclerotic stenosis (ICAS) vessels and evaluate the predictive value of serum nerve injury-induced protein 1 (NINJ1) and matrix metalloproteinase 9 (MMP9) for the vulnerability of intracranial atherosclerotic plaques.

**Methods:**

This study included symptomatic intracranial atherosclerotic stenosis (sICAS) patients who underwent high-resolution vessel wall imaging (HR-VWI) and healthy individuals. Patients were divided into non-enhancement/enhancement, moderate/severe stenosis, and positive/negative remodeling groups. Multivariate logistic regression and receiver operating characteristic (ROC) curve analyses were used to evaluate the predictive value of NINJ1 and MMP9 for plaque enhancement, severe stenosis, and positive remodeling.

**Results:**

NINJ1 and MMP9 levels were higher in the plaque enhancement group compared to the non-enhancement group (107.04 vs. 93.49, *p* = 0.001; 245.35 vs. 227.16, *p* = 0.002) and were independent risk factors for plaque enhancement (OR: 1.036, *p* = 0.003; OR: 1.022, *p* = 0.008). The area under the curve (AUC) for predicting plaque enhancement by NINJ1 and MMP9 were 0.676 and 0.667, respectively, and the combined AUC was 0.740. In the severe stenosis group, NINJ1 and MMP9 levels were also higher than in the moderate stenosis group (106.28 vs. 94.54, *p* = 0.006; 243.88 vs. 229.38, *p* = 0.014), with both being independent risk factors (OR: 1.027, *p* = 0.012; OR: 1.017, *p* = 0.027). The AUC for predicting severe stenosis by NINJ1 and MMP9 were 0.652 and 0.646, respectively, and the combined AUC was 0.686. For the positive remodeling group, NINJ1 and MMP9 levels were significantly elevated (108.73 vs. 97.27, *p* = 0.007; 248.36 vs. 230.42, *p* = 0.002), and both were independent risk factors (OR: 1.026, *p* = 0.015; OR: 1.023, *p* = 0.004). The AUC for predicting positive remodeling by NINJ1 and MMP9 were 0.642 and 0.672, respectively, and the combined AUC was 0.722.

**Conclusion:**

NINJ1 and MMP9 can serve as independent predictors factors for intracranial atherosclerotic plaque enhancement, severe stenosis, and positive remodeling. NINJ1 and MMP9 have the potential to be serum biomarkers for the vulnerability of intracranial atherosclerotic plaques.

## 1 Introduction

Atherosclerosis is a chronic inflammatory disease affecting blood vessels and can lead to serious complications due to persistent inflammatory processes. Depending on the affected vessels, it can result in cardiovascular diseases, stroke, and others (1). Intracranial atherosclerotic disease is considered one of the leading causes of stroke worldwide (2). According to the Warfarin versus Aspirin Therapy for Symptomatic Intracranial Arterial Stenosis study, symptomatic intracranial atherosclerotic stenosis (sICAS) refers to ischemic stroke and/or transient ischemic attack (TIA) occurring within the past 3 months, with intracranial arterial stenosis (narrowing 50 to 99%) caused by atherosclerosis, the lesion being located within the arterial supply area, or TIA symptoms matching the neurologic deficit in the corresponding brain tissue supplied by the affected artery (3). A single-center study reported that the prevalence of intracranial atherosclerotic stenosis accounted for 33 to 67% of stroke and TIA cases in China and other Asian countries (4). As is well known, inflammation plays a crucial role in the formation of atherosclerosis and the progression of atherosclerotic plaques (5, 6). Nerve injury-induced protein 1 (NINJ1) is a cell surface adhesion protein. Under the action of injury and inflammatory factors, leukocytes (monocytes, neutrophils, etc.) are activated, leading to an increase in NINJ1 expression on their surface. Leukocytes expressing NINJ1 strongly adhere to endothelial cells and other leukocytes through homophilic or heterophilic patterns and migrate to the inflammation site, further enhancing the inflammatory response (7). Matrix metalloproteinase 9 (MMP9) is a zinc-dependent endopeptidase family member responsible for tissue remodeling and the degradation of extracellular matrix (ECM) proteins. It can degrade the fibrous cap of atherosclerotic plaques, promote the migration of inflammatory cells to the atherosclerotic site and promote the progression of atherosclerosis (8). High-resolution blood vessel wall imaging (HR-VWI) can visualize the intracranial arterial wall and provide imaging results such as plaque enhancement degree and remodeling type (9, 10). Although HR-VWI allows for the precise assessment of vulnerable plaques, its clinical application is constrained by factors such as prolonged acquisition time and high cost. Given that blood samples are more readily obtainable, this study aims to identify serum biomarkers closely associated with HR-VWI characteristics of atherosclerosis. Specifically, the association between serum NINJ1 and MMP9 and HR-VWI features warrants further investigation.

## 2 Materials and methods

### 2.1 Study population

This study collected patients diagnosed with symptomatic intracranial atherosclerotic stenosis (sICAS) and underwent HR-VWI in the First Affiliated Hospital of Xinxiang Medical University between October 2023 and August 2024 as the study group. Healthy individuals undergoing routine physical examinations during the same period were selected as the control group. Inclusion criteria for the study group: (1) Age between 18 and 80 years. (2) Patients with symptomatic intracranial atherosclerotic stenosis who were enrolled within 7 days of symptom onset. (3) Underwent high-resolution vessel wall imaging within 7 days after symptom onset, and imaging results indicated the presence of culprit plaques. (4) Imaging confirmed sICAS patients with intracranial responsible artery stenosis ≥50%. (5) At least one risk factor for atherosclerosis, such as hypertension, hyperlipidemia, diabetes, coronary artery disease, obesity or smoking. Exclusion criteria for the study group: (1) Non-symptomatic intracranial atherosclerotic stenosis patients. (2) Responsible artery and culprit plaque located in the extracranial segment of the internal carotid artery or vertebral artery. (3) Patients with occlusion of the responsible artery. (4) Intracranial artery stenosis caused by non-atherosclerotic diseases, such as vasculitis, arterial dissection, moyamoya disease, etc. (5) Patients with atrial fibrillation, asthma, pulmonary fibrosis, inflammatory bowel disease, malignancies, severe infections, etc. (6) Poor image quality or missing data. Inclusion criteria for the control group: (1) Age between 18 and 80 years. (2) No history of cerebrovascular disease. (3) No history of atherosclerosis-related diseases, such as hypertension, diabetes mellitus, coronary artery disease, peripheral arterial disease, or hyperlipidemia. (4) No severe systemic diseases, such as malignancies, autoimmune diseases, or infectious diseases. (5) No history of smoking or alcohol consumption. (6) No recent infections or inflammatory conditions. (7) No history of long-term medication use. Exclusion criteria for the control group: (1) Presence of neurological diseases, such as stroke or multiple sclerosis. (2) Presence of high-risk factors for atherosclerosis, such as hypertension, hyperlipidemia, diabetes mellitus, or coronary artery disease. (3) Recent history of surgery or trauma. This study was approved by the Ethics Committee of the First Affiliated Hospital of Xinxiang Medical University (No. EC-024-291).

### 2.2 Data collection

We collected baseline data of the patients, including age, gender, body mass index (BMI), smoking history, drinking history, hypertension history, diabetes history, history of cerebrovascular disease, and coronary artery disease. Laboratory test indicators included total cholesterol (TC), triglycerides (TG), low-density lipoprotein cholesterol (LDL), high-density lipoprotein cholesterol (HDL), glycated hemoglobin (HbA1c), homocysteine, fibrinogen, white blood cell count and neutrophil count.

### 2.3 Detection of NINJ1 and MMP9

Venous blood samples were collected from patients within 48 h of admission and from healthy controls in the health examination center. The samples were immediately stored at −4°C after collection. After overnight storage at −4°C, the blood samples were centrifuged at 3000 rpm for 10 min and the resulting serum was frozen at −80°C until laboratory analysis. Prior to testing, the samples were thawed, and serum levels of NINJ1 and MMP9 were measured using enzyme-linked immunosorbent assay (ELISA) kits for NINJ1 and MMP9 (Jiangsu Meiyu Immuno Industry Co., Ltd.), following the manufacturer’s instructions to assess the serum levels in both the study and control groups.

### 2.4 Magnetic resonance imaging

Each patient underwent routine cranial magnetic resonance imaging (MRI) and high-resolution blood vessel wall imaging (HR-VWI) scans. The MRI included diffusion-weighted imaging (DWI), three-dimensional time-of-flight magnetic resonance angiography (3D-TOF MRA), T1-weighted imaging (T1WI), and T2-weighted imaging (T2WI). For high-resolution MRI, a three-dimensional isotropic turbo spin echo T1-weighted volume scan (3D T1-weighted, 3DT1W-VISTA) was performed first. After imaging, gadobutrol (0.1 mmol/kg) was injected, followed by a second 3D T1W-VISTA scan. The scanning parameters for each sequence are as follows: (1) DWI Sequence: TR 2,194 ms, TE 86 ms, FOV 230 mm × 230 mm × 109 mm, slice thickness 5 mm, voxel size 1.5 mm × 1.89 mm × 5 mm, matrix size 152 × 122 × 17, interslice gap 1.5 mm, scan time 33 s; (2) 3D-TOF MRA Sequence: TR 19 ms, TE 3.5 ms, FOV 200 mm × 158 mm × 89 mm, slice thickness 1.2 mm, voxel size 0.65 mm × 0.94 mm × 1.2 mm, matrix size 308 × 168 × 148, interslice gap −0.6 mm, scan time 2 min 52 s; (3) T1WI Sequence: TR 2373 ms, TE 20 ms, FOV 230 mm × 189 mm × 109 mm, slice thickness 5 mm, voxel size 0.8 mm × 1.05 mm × 5 mm, matrix size 288 × 178 × 17, interslice gap 1.5 mm, scan time 1 min 30 s; (4) T2WI Sequence: TR 2756 ms, TE 105 ms, FOV 230 mm × 230 mm × 109 mm, slice thickness 5 mm, voxel size 0.95 mm × 0.95 mm × 5 mm, matrix size 244 × 244 × 17, interslice gap 1.5 mm, scan time 44 s; (5) 3D T1W-VISTA Sequence: TR 600 ms, TE 31 ms, FOV 250 mm × 161 mm × 60 mm, slice thickness 0.6 mm, voxel size 0.8 mm × 0.8 mm × 0.8 mm, matrix size 312 × 201 × 150, interslice spacing −0.4 mm, and scan time 3 min 36 s.

### 2.5 Image post-processing and analysis

**Figure 1:**
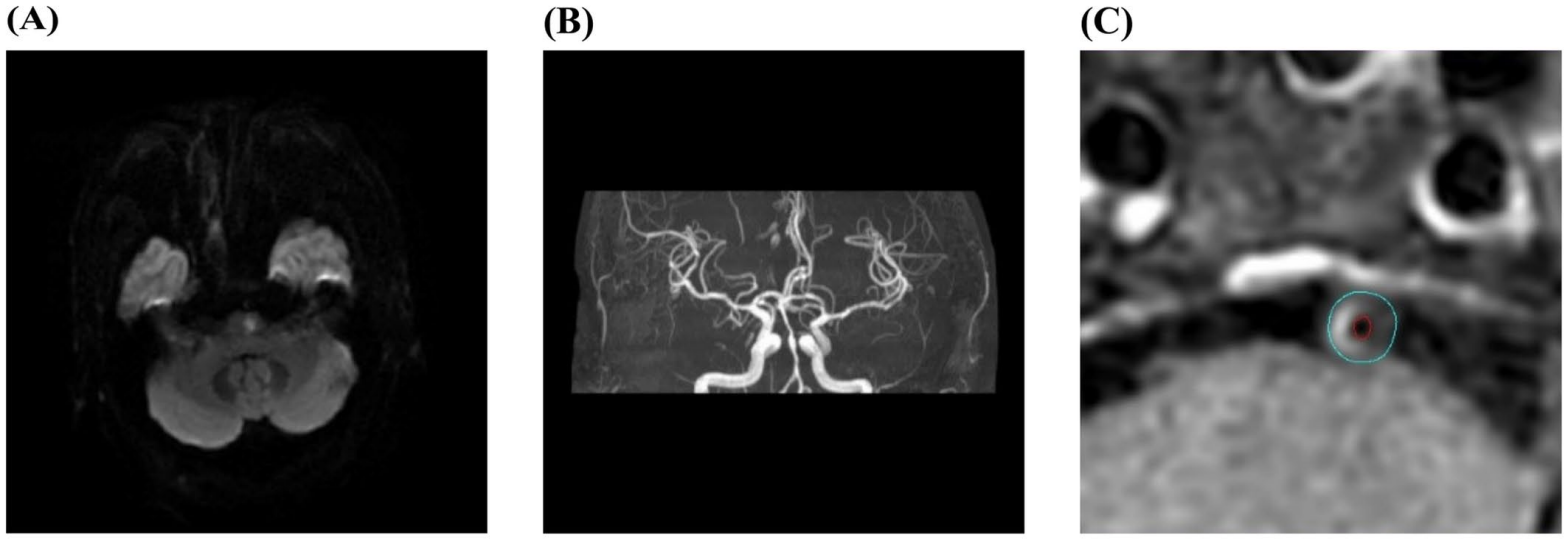
Typical images of contrast-enhanced high-resolution vessel wall imaging in a sICAS patient. **(A–C)** Male, 56 years old, with right-sided limb weakness for 1 day. DWI showed acute infarction in the left pons **(A)**; MRA showed severe stenosis of the basilar artery **(B)**; HR-VWI enhanced image showed an enhanced plaque in the basilar artery. The blue line outlined the vessel wall area, and the red line outlined the lumen area **(C)**.

The stenotic vessel causing cerebral infarction or TIA was designated as the responsible vessel and the atherosclerotic plaque formed in the responsible vessel was defined as the culprit plaque. If multiple plaques were present in the same vascular territory, the narrowest plaque was selected for analysis. All images were independently evaluated by two experienced radiologists. For HR-VWI sequences, reconstruction was performed using the VesselExplorer2 workstation (Beijing Qingying Huakang Technology Co., Ltd.), producing coronal, sagittal and axial images. Axial images perpendicular to the long axis of the responsible vessel were selected for analysis. The slices containing the responsible vessel were magnified by 400%, and the outer contour of the vessel wall and the inner contour of the lumen were manually outlined. The software automatically measured the vessel area, lumen area and wall area. The narrowest plaque was selected as the culprit plaque for measurement. When selecting reference sites for vessel area and lumen area, priority was given to the proximal segment of the stenotic lumen without significant narrowing, followed by the distal segment without significant narrowing. The following formulas were used for calculating relevant indicators: vascular stenosis rate = (1-lumen area at stenosis/lumen area at reference) * 100%, wall area = blood vessel area - lumen area, 50% ≤ stenosis rate < 70% is defined as moderate stenosis, 70% ≤ stenosis rate ≤ 99% is defined as severe stenosis; remodeling index (RI) = vessel area at stenosis/vessel area at reference, RI ≥ 1.05 indicates positive remodeling, 0.95 < RI < 1.05 indicates no remodeling, RI ≤ 0.95 indicates negative remodeling (11); normalized wall index (NWI) = wall area at stenosis/vessel area at stenosis. The degree of plaque enhancement was assessed using contrast-enhanced T1W-VISTA images: Grade 0: plaque enhancement is equal to or less than that of the adjacent normal vessel wall without plaque, indicating no enhancement; Grade 1: plaque enhancement is greater than that of the adjacent normal vessel wall but less than that of the pituitary stalk, indicating mild enhancement; Grade 2: plaque enhancement is equal to or greater than that of the pituitary stalk, indicating significant enhancement (12). Grades 1 and 2 were classified as enhanced plaques. Representative imaging data of typical sICAS patients are shown in Figure 1.

### 2.6 Statistical analysis

Statistical analysis was performed using SPSS 27.0 software. Categorical data were presented as percentages, and intergroup comparisons were conducted using the Chi-square test. The normality of continuous variables, including NINJ1 and MMP9 levels, was assessed using the Shapiro–Wilk test. Normally distributed continuous data were presented as mean ± standard deviation (x̅ ± s), and comparisons between two groups were performed using the *t*-test. For non-normally distributed data, the median and interquartile range (P25-P75) were used, with comparisons between two groups made using the Mann–Whitney U test. Significant indicators from univariate analysis were further analyzed using multivariate logistic regression. Receiver operating characteristic (ROC) curve analysis was employed to assess predictive factors and calculate the area under the curve (AUC). The *p*-value of <0.05 was considered statistically significant.

## 3 Results

### 3.1 Comparison between sICAS patients and healthy individuals

A total of 110 patients diagnosed with sICAS and undergoing high-resolution intracranial artery vessel wall imaging in the First Affiliated Hospital of Xinxiang Medical University from October 2023 to August 2024 were included in this study, along with 30 healthy individuals undergoing routine health checks. The normality of serum NINJ1 and MMP9 levels was assessed using the Shapiro–Wilk test. The results indicated that NINJ1 and MMP9 levels were normally distributed in both the sICAS and control groups (*p* > 0.05 for both). There were no statistically significant differences in age and gender between the two groups (*p* > 0.05). However, the serum levels of NINJ1 (102.12 vs. 83.58, *p* < 0.001) and MMP9 (238.74 vs. 152.12, *p* < 0.001) were significantly higher in the sICAS patients compared to the healthy controls (see Table 1).

**Table 1:** Comparison of clinical data between sICAS patients and healthy control group.

Project	Study group (*n* = 110)	Control group (*n* = 30)	*t/Z/χ^2^*	*p-*value
Age (years)	57.77 ± 12.29	55.97 ± 8.48	−0.756	0.451
Male	61 (55.5%)	19 (63.3%)	0.597	0.440
NINJ1 (ng/ml)	102.12 ± 21.49	83.58 ± 28.98	−3.867	<0.001
MMP9 (μg/L)	238.74 ± 29.70	152.12 ± 50.13	−12.018	<0.001

NINJ1, nerve injury-induced protein 1; MMP9, matrix metalloproteinase 9.

### 3.2 Comparison of clinical data between the non-enhanced plaque group and the enhanced plaque group

Based on the presence or absence of plaque enhancement, the study population was divided into the non-enhanced plaque group and the enhanced plaque group. The normality of serum NINJ1 and MMP9 levels within each group was assessed using the Shapiro–Wilk test. The results indicated that NINJ1 and MMP9 levels were normally distributed in both the enhanced and non-enhanced plaque groups (*p* > 0.05 for both). Comparison of Baseline Data: There were no significant differences between the two groups in terms of age, gender, BMI, history of hypertension, history of diabetes, history of heart disease, history of cerebrovascular disease, smoking history, and drinking history (*p* > 0.05). Laboratory Test Indicators Comparison: Compared with the non-enhanced plaque group, NINJ1 (107.04 vs. 93.49, *p* = 0.001), MMP9 (245.35 vs. 227.16, *p* = 0.002), and glycosylated hemoglobin (6.92 vs. 6.16, *p* = 0.028) were significantly higher in the enhanced plaque group. No significant differences were found for other indicators between the two groups (*p* > 0.05). Imaging Indicators Comparison: The enhanced plaque group showed significantly higher vascular stenosis degree (78.83 vs. 68.12, *p* < 0.001), NWI (86.77 vs. 76.37, *p* < 0.001) and reconstruction index (1.04 vs. 0.80, *p* = 0.007) compared to the non-enhanced group. There was no significant difference between the two groups in terms of plaque location (*p* > 0.05) (see Table 2).

**Table 2:** Comparison of clinical data between sICAS patients with and without plaque enhancement.

Project	Total (*n* = 110)	Non-enhanced (*n* = 40)	Enhanced (*n* = 70)	*t/Z/χ^2^*	*p-*value
Baseline data					
Age (years)	57.77 ± 12.29	58.15 ± 12.96	57.56 ± 11.98	0.242	0.809
Male	61 (55.5%)	21 (52.5%)	40 (57.1%)	0.222	0.637
BMI (kg/m^2^)	25.71 ± 3.60	25.35 ± 2.79	25.91 ± 3.99	−0.783	0.436
Hypertension	80 (72.7%)	30 (75.0%)	50 (71.4%)	0.164	0.686
Diabetes	38 (34.5%)	12 (30.0%)	26 (37.1%)	0.574	0.449
Heart disease	14 (12.7%)	2 (5.0%)	12 (17.1%)	3.379	0.066
Cerebrovascular disease	54 (49.1%)	18 (45.0%)	36 (51.4%)	0.421	0.516
Smoking history	40 (36.4%)	15 (37.5%)	25 (35.7%)	0.035	0.851
Drinking history	18 (16.4%)	7 (17.5%)	11 (15.7%)	0.059	0.808
Laboratory indicators					
TC (mmol/L)	4.01 (3.44, 4.75)	4.14 (3.36, 4.76)	3.99 (3.47, 4.62)	−0.786	0.432
TG (mmol/L)	1.35 (0.97, 1.75)	1.32 (0.82, 1.57)	1.44 (1.03, 1.84)	−1.488	0.137
LDL (mmol/L)	2.43 ± 0.80	2.26 ± 0.74	2.52 ± 0.82	−1.671	0.098
HDL (mmol/L)	1.03 ± 0.29	1.00 ± 0.26	1.04 ± 0.30	−0.848	0.399
White blood cell (*10^9^/L)	6.73 (5.61, 8.00)	6.33 (5.43, 7.68)	6.98 (5.73, 8.44)	−1.364	0.173
Neutrophil (*10^9^/L)	4.32 (3.45, 5.59)	4.15 (3.47, 5.14)	4.42 (3.41, 5.78)	−0.497	0.619
HbA1c (%)	6.60 (5.48, 7.65)	6.16 (5.15, 7.13)	6.92 (5.70, 7.82)	−2.203	0.028
Homocysteine (μmol/L)	15.05 (11.72, 20.23)	13.90 (11.12, 18.12)	16.16 (12.21, 20.39)	−1.131	0.258
Fibrinogen (mg/dL)	301.80 (264.90, 351.93)	297.30 (248.75, 344.20)	308.70 (271.63, 360.60)	−1.644	0.100
NINJ1 (ng/ml)	102.12 ± 21.49	93.49 ± 19.77	107.04 ± 21.00	−3.325	0.001
MMP9 (μg/L)	238.74 ± 29.70	227.16 ± 28.80	245.35 ± 28.32	−3.205	0.002
Imaging data					
Degree of stenosis (%)	75.75 (65.07, 82.64)	68.12 (57.38, 75.25)	78.83 (72.28, 86.61)	−4.303	<0.001
Plaque location					
Anterior circulation	57 (51.8%)	22 (55.0%)	35 (50.0%)	0.255	0.614
Posterior circulation	53 (48.2%)	18 (45.0%)	35 (50.0%)		
RI	0.89 (0.70, 1.19)	0.80 (0.66, 0.90)	1.04 (0.73, 1.26)	−2.675	0.007
NWI (%)	83.96 (75.37, 88.90)	76.37 (69.88, 82.83)	86.77 (81.03, 90.09)	−4.583	<0.001

BMI, body mass index; TC, total cholesterol; TG, triglycerides; LDL, low-density lipoprotein cholesterol; HDL, high-density lipoprotein cholesterol; HbA1c, glycated hemoglobin; NINJ1, nerve injury-induced protein 1; MMP9, matrix metalloproteinase 9; RI, reconstruction index; NWI, normalized vessel wall index.

### 3.3 Comparison of clinical data between moderate and severe stenosis groups

Based on the degree of stenosis at the most narrowed part of the responsible artery, the study population was divided into moderate and severe stenosis groups. Serum NINJ1 and MMP9 levels in both the moderate and severe stenosis groups were tested for normality using the Shapiro–Wilk test, and the results confirmed that the distributions were normal (*p* > 0.05 for both). Baseline Data Comparison: There were no statistically significant differences in age, gender, BMI, history of hypertension, diabetes, heart disease, cerebrovascular disease, smoking history, and drinking history between the two groups (*p* > 0.05). Laboratory Test Indicators Comparison: The levels of NINJ1 (106.28 vs. 94.54, *p* = 0.006) and MMP9 (243.88 vs. 229.38, *p* = 0.014) were significantly higher in the severe stenosis group compared to the moderate stenosis group, while no significant differences were observed for other indicators between the two groups (*p* > 0.05). Imaging Indicators Comparison: The severe stenosis group had a significantly higher NWI (86.79 vs. 75.11, *p* < 0.001) compared to the moderate stenosis group. The proportion of plaque enhancement was also higher in the severe stenosis group (77.5 vs. 38.5, *p* < 0.001). There was a statistically significant difference in plaque distribution location between the two groups (*p* = 0.013), with plaques predominantly located in the posterior circulation (64.1%) in the moderate stenosis group and in the anterior circulation (60.6%) in the severe stenosis group. There was no statistically significant difference in the reconstruction index (*p* > 0.05) (see Table 3).

**Table 3:** Comparison of clinical data between moderate stenosis group and severe stenosis group in sICAS patients.

Project	Total (*n* = 110)	Moderate stenosis (*n* = 39)	Severe stenosis (*n* = 71)	*t/Z/χ^2^*	*p-*value
Baseline data					
Age (years)	57.77 ± 12.29	58.05 ± 13.35	57.62 ± 11.76	0.175	0.861
Male	61 (55.5%)	22 (56.4%)	39 (54.9%)	0.022	0.881
BMI (kg/m^2^)	25.71 ± 3.60	25.18 ± 3.26	26.00 ± 3.76	−1.148	0.253
Hypertension	80 (72.7%)	27 (69.2%)	53 (74.6%)	0.372	0.542
Diabetes	38 (34.5%)	14 (35.9%)	24 (33.8%)	0.049	0.825
Heart disease	14 (12.7%)	6 (15.4%)	8 (11.3%)	0.384	0.535
Cerebrovascular disease	54 (49.1%)	18 (46.2%)	36 (50.7%)	0.209	0.648
Smoking history	40 (36.4%)	16 (41.0%)	24 (33.8%)	0.568	0.451
Drinking history	18 (16.4%)	6 (15.4%)	12 (16.9%)	0.042	0.837
Laboratory indicators					
TC (mmol/L)	4.01 (3.44, 4.75)	4.14 (3.80, 4.76)	3.98 (3.24, 4.75)	−1.659	0.097
TG (mmol/L)	1.35 (0.97, 1.75)	1.37 (1.02, 1.79)	1.34 (0.88, 1.74)	−0.303	0.762
LDL (mmol/L)	2.43 ± 0.80	2.41 ± 0.94	2.44 ± 0.71	−0.211	0.834
HDL (mmol/L)	1.03 ± 0.29	1.08 ± 0.29	1.00 ± 0.28	1.371	0.173
White blood cell (*10^9^/L)	6.73 (5.61, 8.00)	6.64 (5.48, 7.68)	6.73 (5.70, 8.21)	−0.765	0.444
Neutrophil (*10^9^/L)	4.32 (3.45, 5.59)	4.33 (3.34, 5.32)	4.30 (3.60, 5.72)	−0.481	0.630
HbA1c (%)	6.60 (5.48, 7.65)	6.54 (5.56, 7.62)	6.63 (5.45, 7.72)	−0.250	0.803
Homocysteine (μmol/L)	15.05 (11.72, 20.23)	14.87 (11.20, 22.15)	15.10 (11.77, 20.11)	−0.384	0.701
Fibrinogen (mg/dL)	301.80 (264.90, 351.93)	296.00 (251.30, 360.60)	304.20 (272.00, 350.10)	−1.412	0.158
NINJ1 (ng/ml)	102.12 ± 21.49	94.54 ± 18.81	106.28 ± 21.86	−2.828	0.006
MMP9 (μg/L)	238.74 ± 29.70	229.38 ± 28.97	243.88 ± 29.02	−2.509	0.014
Imaging data					
Degree of enhancement					
Without enhancement	40 (36.4%)	24 (61.5%)	16 (22.5%)	16.549	<0.001
With enhancement	70 (63.6%)	15 (38.5%)	55 (77.5%)		
Plaque location					
Anterior circulation	57 (51.8%)	14 (35.9%)	43 (60.6%)	6.134	0.013
Posterior circulation	53 (48.2%)	25 (64.1%)	28 (39.4%)		
RI	0.89 (0.70, 1.19)	0.89 (0.81, 1.29)	0.89 (0.67, 1.13)	−1.850	0.064
NWI (%)	83.96 (75.37, 88.90)	75.11 (66.61, 82.18)	86.79 (81.30, 90.49)	−5.271	<0.001

BMI, body mass index; TC, total cholesterol; TG, triglycerides; LDL, low-density lipoprotein cholesterol; HDL, high-density lipoprotein cholesterol; HbA1c, glycated hemoglobin; NINJ1, nerve injury-induced protein 1; MMP9, matrix metalloproteinase 9; RI, reconstruction index; NWI, normalized vessel wall index.

### 3.4 Comparison of clinical data between positive and negative remodeling groups

Based on the remodeling type of the responsible artery, the study population was divided into positive and negative remodeling groups, excluding patients with no remodeling. Normality testing of serum NINJ1 and MMP9 levels using the Shapiro–Wilk test demonstrated that both indicators followed a normal distribution in the positive and negative remodeling groups (*p* > 0.05 for both). Baseline Data Comparison: There were no statistically significant differences in any of the indicators between the two groups (*p* > 0.05). Laboratory Test Indicators Comparison: The levels of NINJ1 (108.73 vs. 97.27, *p* = 0.007) and MMP9 (248.36 vs. 230.42, *p* = 0.002) were significantly higher in the positive remodeling group compared to the negative remodeling group, while there were no significant differences in other indicators between the two groups (*p* > 0.05). Imaging Indicators Comparison: The degree of stenosis was significantly lower in the positive remodeling group (72.38 vs. 75.91, *p* = 0.034) compared to the negative remodeling group. The NWI (86.93 vs. 80.24, *p* < 0.001) was significantly higher in the positive remodeling group. The proportion of plaque enhancement was higher in the positive remodeling group (79.5 vs. 49.2, *p* = 0.002) compared to the negative remodeling group. There was a statistically significant difference in plaque distribution location between the two groups (*p* = 0.026). In the positive remodeling group, plaques were more frequently located in the posterior circulation (61.4%), whereas in the negative remodeling group, plaques were more commonly found in the anterior circulation (60.7%) (see Table 4).

**Table 4:** Comparison of clinical data between positive remodeling group and negative remodeling group in sICAS patients.

Project	Total (*n* = 105)	Positive remodeling (*n* = 44)	Negative remodeling (*n* = 61)	*t/Z/χ^2^*	*p-*value
Baseline data					
Age (years)	58.14 ± 11.74	55.89 ± 12.66	59.77 ± 10.85	1.687	0.095
Male	60 (57.1%)	29 (65.9%)	31 (50.8%)	2.377	0.123
BMI (kg/m^2^)	25.66 ± 3.51	25.85 ± 3.91	25.51 ± 3.23	−0.486	0.628
Hypertension	75 (71.4%)	31 (70.5%)	44 (72.1%)	0.035	0.851
Diabetes	36 (34.3%)	15 (34.1%)	21 (34.4%)	0.001	0.972
Heart disease	14 (13.3%)	7 (15.9%)	7 (11.5%)	0.435	0.510
Cerebrovascular disease	51 (48.6%)	24 (54.5%)	27 (44.3%)	1.082	0.298
Smoking history	40 (38.1%)	19 (43.2%)	21 (34.4%)	0.831	0.362
Drinking history	18 (17.1%)	7 (15.9%)	11 (18.0%)	0.081	0.776
Laboratory indicators					
TC (mmol/L)	4.00 (3.40, 4.68)	3.96 (3.27, 4.48)	4.11 (3.43, 4.76)	−0.935	0.350
TG (mmol/L)	1.35 (0.97, 1.75)	1.40 (1.02, 1.96)	1.34 (0.87, 1.72)	−0.909	0.363
LDL (mmol/L)	2.41 ± 0.81	2.47 ± 0.79	2.37 ± 0.82	−0.653	0.515
HDL (mmol/L)	1.02 ± 0.29	1.05 ± 0.33	1.00 ± 0.25	−0.827	0.410
White blood cell (*10^9^/L)	6.73 (5.58, 8.04)	7.09 (5.65, 9.33)	6.64 (5.53, 7.66)	−1.081	0.280
Neutrophil (*10^9^/L)	4.33 (3.46, 5.59)	4.40 (3.36, 6.40)	4.33 (3.74, 5.16)	−0.127	0.899
HbA1c (%)	6.54 (5.47, 7.67)	6.56 (5.59, 7.73)	6.54 (5.39, 7.55)	−0.425	0.671
Homocysteine (μmol/L)	15.00 (11.70, 20.19)	15.18 (12.37, 23.73)	14.87 (11.29, 18.93)	−1.224	0.221
Fibrinogen (mg/dL)	301.24 (264.00, 353.75)	300.50 (254.90, 345.15)	301.74 (268.70, 360.70)	−0.396	0.692
NINJ1 (ng/ml)	102.07 ± 21.74	108.73 ± 19.97	97.27 ± 21.84	−2.750	0.007
MMP9 (μg/L)	237.93 ± 29.21	248.36 ± 28.05	230.42 ± 27.89	−3.245	0.002
Imaging data					
Degree of stenosis (%)	75.73 (64.70, 82.53)	72.38 (55.40, 81.79)	75.91 (67.61, 85.38)	−2.124	0.034
Degree of enhancement					
Without enhancement	40 (38.1%)	9 (20.5%)	31 (50.8%)	9.994	0.002
With enhancement	65 (61.9%)	35 (79.5%)	30 (49.2%)		
Plaque location					
Anterior circulation	54 (51.4%)	17 (38.6%)	37 (60.7%)	4.962	0.026
Posterior circulation	51 (48.6%)	27 (61.4%)	24 (39.3%)		
NWI (%)	83.66 (75.18, 88.73)	86.93 (81.92, 90.94)	80.24 (72.98, 86.65)	−3.475	<0.001

BMI, body mass index; TC, total cholesterol; TG, triglycerides; LDL, low-density lipoprotein cholesterol; HDL, high-density lipoprotein cholesterol; HbA1c, glycated hemoglobin; NINJ1, nerve injury-induced protein 1; MMP9, matrix metalloproteinase 9; NWI, normalized vessel wall index.

### 3.5 Multivariate logistic regression analysis

Laboratory test indicators that showed *p* < 0.05 in univariate analysis (see Tables 2–4) were included in the multivariate regression analysis. The results indicated that elevated levels of NINJ1 and MMP9 were independent risk factors for plaque enhancement (OR: 1.036, 95% CI: 1.012–1.060, *p* = 0.003; OR: 1.022, 95% CI: 1.006–1.038, *p* = 0.008; see Table 5), for severe stenosis (OR: 1.027, 95% CI: 1.006–1.049, *p* = 0.012; OR: 1.017, 95% CI: 1.002–1.032, *p* = 0.027; see Table 6), and for positive vascular remodeling (OR: 1.026, 95% CI: 1.005–1.048, *p* = 0.015; OR: 1.023, 95% CI: 1.007–1.040, *p* = 0.004; see Table 7).

**Table 5:** Logistic regression analysis of factors influencing plaque enhancement in sICAS patients.

Project	OR	95% CI	*p-*value
NINJ1	1.036	1.012–1.060	0.003
MMP9	1.022	1.006–1.038	0.008
HbA1c	1.386	0.995–1.930	0.053

NINJ1, nerve injury-induced protein 1; MMP9, matrix metalloproteinase 9; HbA1c, glycated hemoglobin; OR, odds ratio; CI, confidence interval.

**Table 6:** Logistic regression analysis of factors influencing the degree of stenosis in sICAS patients.

Project	OR	95% CI	*p-*value
NINJ1	1.027	1.006–1.049	0.012
MMP9	1.017	1.002–1.032	0.027

NINJ1, nerve injury-induced protein 1; MMP9, matrix metalloproteinase 9; OR, odds ratio; CI, confidence interval.

**Table 7:** Logistic regression analysis of factors influencing positive remodeling in sICAS patients.

Project	OR	95% CI	*p-*value
NINJ1	1.026	1.005–1.048	0.015
MMP9	1.023	1.007–1.040	0.004

NINJ1, nerve injury-induced protein 1; MMP9, matrix metalloproteinase 9; OR, odds ratio; CI, confidence interval.

### 3.6 ROC curve analysis

**Figure 2:**
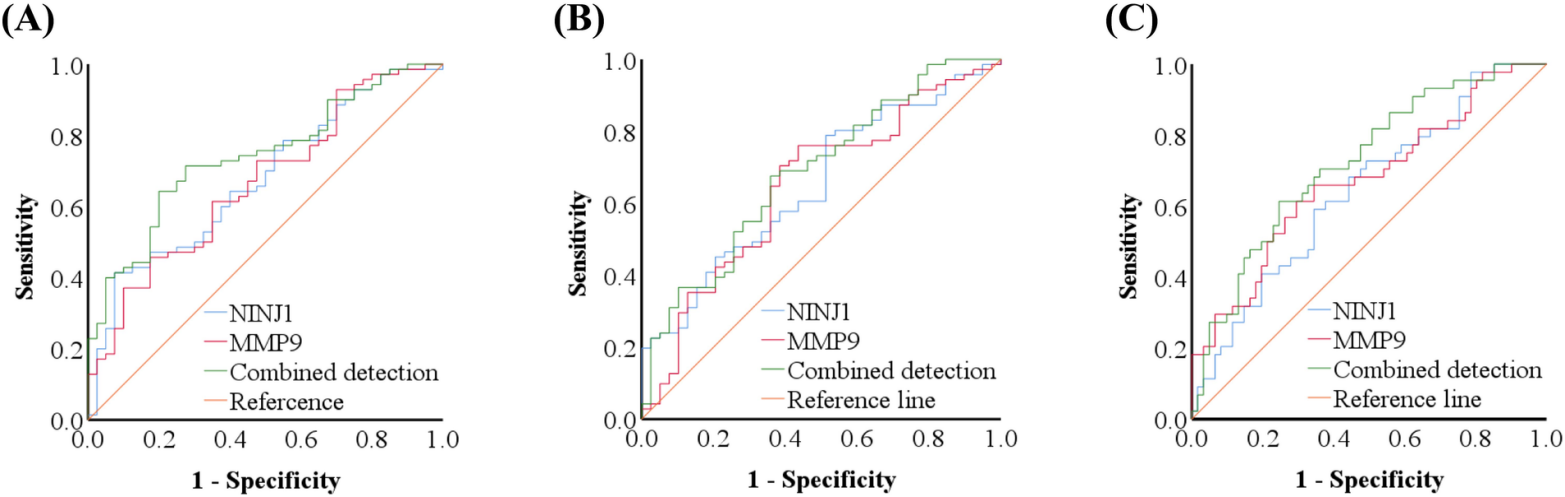
ROC curve of NINJ1 and MMP9 in predicting high-resolution vessel wall imaging features in sICAS patients. ROC curve of NINJ1 and MMP9 in predicting plaque enhancement in sICAS patients **(A)**; ROC curve of NINJ1 and MMP9 in predicting severe stenosis in sICAS patients **(B)**; ROC curve of NINJ1 and MMP9 in predicting positive remodeling in sICAS patients **(C)**.

ROC curve analysis was conducted to evaluate the predictive value of NINJ1 and MMP9 for plaque enhancement, severe stenosis and positive vascular remodeling. The AUC for NINJ1 in predicting plaque enhancement was 0.676, followed by MMP9 with an AUC of 0.667, and the combined prediction of both had an AUC of 0.740. The optimal cutoff value for NINJ1 predicting plaque enhancement was 111.75 ng/mL, with a sensitivity of 41.40% and specificity of 92.50%. The optimal cutoff value for MMP9 was 251.11 μg/L, with a sensitivity of 45.70% and specificity of 82.50% (see Figure 2 and Table 8).

**Table 8:** ROC curve analysis results of the predictive value of NINJ1 and MMP9 for plaque enhancement in sICAS patients.

Project	AUC (95% CI)	Cut-off value	Sensitivity (%)	Specificity (%)	*p-*value
NINJ1	0.676 (0.575–0.778)	111.75 ng/mL	41.40	92.50	0.002
MMP9	0.667 (0.564–0.770)	251.11 μg/L	45.70	82.50	0.004
Combined detection	0.740 (0.648–0.833)	-	64.30	80.00	<0.001

ROC, receiver operating characteristic; AUC, the area under the ROC curve; CI, confidence interval; NINJ1, nerve injury-induce d protein 1, MMP9, matrix metalloproteinase 9.

The AUC for NINJ1 in predicting severe vascular stenosis was 0.652, followed by MMP9 with an AUC of 0.646, and the combined prediction of both had an AUC of 0.686. The optimal cutoff value for NINJ1 in predicting severe stenosis was 92.35 ng/mL, with a sensitivity of 78.90% and specificity of 48.70%. The optimal cutoff value for MMP9 was 229.26 μg/L, with a sensitivity of 76.10% and specificity of 56.40% (see Figure 2 and Table 9).

**Table 9:** ROC curve analysis results of the predictive value of NINJ1 and MMP9 for severe stenosis in sICAS patients.

Project	AUC (95% CI)	Cut-off value	Sensitivity (%)	Specificity (%)	*p-*value
NINJ1	0.652 (0.548–0.756)	92.35 ng/mL	78.90	48.70	0.009
MMP9	0.646 (0.538–0.755)	229.26 μg/L	76.10	56.40	0.011
Combined detection	0.686 (0.583–0.789)	-	67.60	64.10	0.001

ROC, receiver operating characteristic; AUC, the area under the ROC curve; CI, confidence interval; NINJ1, nerve injury-induce d protein 1, MMP9, matrix metalloproteinase 9.

The AUC for NINJ1 in predicting positive vascular remodeling was 0.642, while the AUC for MMP9 was 0.672, and the combined prediction of both had an AUC of 0.722. The optimal cutoff value for NINJ1 in predicting positive vascular remodeling was 104.29 ng/mL, with a sensitivity of 59.10% and specificity of 65.60%. The optimal cutoff value for MMP9 was 243.37 μg/L, with a sensitivity of 61.40% and specificity of 70.50% (see Figure 2 and Table 10).

**Table 10:** ROC curve analysis results of the predictive value of NINJ1 and MMP9 for positive remodeling in sICAS patients.

Project	AUC (95% CI)	Cut-off value	Sensitivity (%)	Specificity (%)	*p-*value
NINJ1	0.642 (0.536–0.748)	104.29 ng/mL	59.10	65.60	0.013
MMP9	0.672 (0.566–0.777)	243.37 μg/L	61.40	70.50	0.003
Combined detection	0.722 (0.625–0.820)	-	61.40	75.40	<0.001

ROC, receiver operating characteristic; AUC, the area under the ROC curve; CI, confidence interval; NINJ1, nerve injury-induce d protein 1, MMP9, matrix metalloproteinase 9.

## 4 Discussion

This study is the first to reveal the correlation between NINJ1, MMP9 and plaque enhancement, arterial stenosis, and vascular remodeling. Furthermore, NINJ1 and MMP9 are independent risk factors for plaque enhancement, arterial stenosis, and positive vascular remodeling.

Atherosclerosis is widely prevalent in both intracranial and extracranial vessels and is a major cause of ischemic cerebrovascular disease. The plaques formed by atherosclerosis are categorized into stable and vulnerable plaques (13). Vulnerable plaques are defined as any type of plaque that has a high likelihood of thrombotic complications and rapid progression. Their characteristics include a thin fibrous cap (<65 μm), a large lipid pool (>40% of the plaque), infiltrating inflammatory cells (particularly macrophages), positive remodeling, neovascularization, and intraplaque hemorrhage (14). Local plaque stability is associated with cerebral ischemic events. A previous study found that culprit plaques exhibit significant enhancement within 4 weeks after acute stroke, whereas the degree of plaque enhancement gradually decreases over time in the subacute (4–12 weeks) and chronic stages (beyond 12 weeks) (15). The degree of plaque enhancement may vary at different stages. In the early phase, low-level inflammation may not produce obvious enhancement. As inflammatory activation intensifies, neovascularization increases, and plaque instability worsens, plaques tend to exhibit marked enhancement. With the resolution of inflammation and plaque stabilization, the enhancement signal progressively diminishes. Therefore, enhancement signals can dynamically reflect the activity and instability of plaques. Investigating serum biomarkers associated with plaque enhancement is thus of great clinical significance. The individualized evaluation of vulnerable plaques has become a research direction in the field of symptomatic intracranial and extracranial atherosclerotic diseases. Currently, the primary method for assessing arterial plaques is high-resolution vessel wall imaging (HR-VWI), but this examination has limitations such as being time-consuming and expensive. As a result, many studies are attempting to identify alternative biomarkers to replace HR-VWI.

NINJ1 is a cell surface adhesion protein that contains an extracellular adhesion domain and two transmembrane regions. The key adhesion domain of NINJ1 is located between proline 26 and asparagine 37 in the extracellular NH2-terminal domain, a 12-residue region composed of clusters of tryptophan and arginine residues. Its function is to mediate cell adhesion through homophilic or heterophilic interactions. NINJ1 was named because it was initially discovered in damaged nerve terminals, but it is expressed in various cell types, including epithelial cells, leukocytes, pericytes, and others (7, 16). NINJ1 plays an important role in atherosclerosis, with existing studies showing that NINJ1 expression is elevated in various inflammatory cells involved in atherosclerosis. Under conditions such as endoplasmic reticulum stress, oxidative stress, and activation of the NF-kB inflammatory pathway, NINJ1 expression increases. NINJ1 promotes the adhesion of inflammatory cells to endothelial cells and their transendothelial migration to the sites of atherosclerosis, thereby facilitating the progression of atherosclerosis (17). Additionally, NINJ1 is involved in the formation of neovascularization, generating vascular nutritive tubes at the shoulders of atherosclerotic plaques to provide nutrients to the plaques. These nutritive blood vessels increase as the plaque progresses. However, under inflammatory and hypoxic conditions, the endothelial integrity of these vessels is impaired, which manifests as contrast agent extravasation and plaque enhancement on HR-VWI examinations (18–20). Clinical studies have demonstrated that serum NINJ1 levels are significantly elevated in patients with coronary atherosclerotic heart disease and are associated with the severity of coronary stenosis, serving as an independent risk factor for cardiovascular disease (21). In addition, high NINJ1 levels have been linked to a higher risk of large-artery atherosclerotic acute ischemic stroke (22). However, research on the association between NINJ1 and intracranial atherosclerosis and plaque vulnerability remains limited. In this study, high-resolution vessel wall imaging (HR-VWI) was utilized to investigate the relationship between serum NINJ1 levels and intracranial atherosclerotic plaque vulnerability features in patients with symptomatic intracranial atherosclerotic stenosis (sICAS).

This study found that serum NINJ1 levels in sICAS patients were significantly higher than those in healthy controls, consistent with the findings reported by Dong et al. (22). In sICAS patients, NINJ1 is an independent risk factor for plaque enhancement and severe stenosis. This may be because NINJ1 promotes the adhesion of monocytes and macrophages to endothelial cells, leading to endothelial dysfunction. This process allows more monocytes to migrate from the endothelial cells to the subintimal layer, where they differentiate into macrophages. These macrophages then take up oxidized low-density lipoprotein via “scavenger” receptors, resulting in foam cell formation and exacerbating atherosclerosis. Additionally, NINJ1 mediates cell membrane rupture, promoting the death of inflammatory cells, releasing damage-associated molecular patterns that promote inflammation and further accelerate atherosclerosis (23).Histopathological and contrast-enhanced magnetic resonance studies of carotid plaques have found that plaque enhancement in high-resolution vessel wall imaging is often associated with inflammation and neovascularization within the plaque (24). This is consistent with the role of NINJ1, suggesting that NINJ1 may be associated with plaque enhancement through its involvement in plaque inflammation and promotion of neovascularization.

This study also found that NINJ1 is related to vascular remodeling, likely due to its pro-inflammatory effect, which can promote the formation of atherosclerotic plaques, increasing plaque load. Regarding the occurrence of vascular remodeling, it is currently believed to be a vascular adaptive response to hemodynamics. The diameter of the vessel is regulated by blood flow, while the thickness of the vessel wall changes according to the tension generated by blood pressure. Arterial blood flow and blood pressure cause two major types of hemodynamic stress: wall shear stress and wall tensile stress. Under physiological conditions, the vascular wall adapts well to these stresses and alters its structure to accommodate changes in these stresses. For example, chronic changes in blood flow lead to changes in arterial diameter, while increased blood pressure leads to thickening of the arterial wall (25). However, when the initial structural changes in the artery caused by hemodynamics are “consolidated” through the involvement of extracellular matrix proteins, arterial remodeling occurs (26). Arteries affected by atherosclerotic lesions can exhibit either positive or negative remodeling. Positive remodeling is considered a compensatory outward expansion of the arterial wall to maintain lumen patency in the presence of plaque formation, while lumen compromise occurs mainly when plaque expansion exceeds the limit of compensatory remodeling. Negative remodeling is defined as adaptive constriction of the vessel, which may exacerbate lumen narrowing (27). Previous studies have shown that positive remodeling is an unsafe form of remodeling, corresponding to higher plaque loads, reflecting low vascular stability and an increased risk of acute ischemic stroke (28). Moreover, sICAS patients have a higher incidence of positive remodeling compared to asymptomatic ICAS patients (29).

Matrix metalloproteinases (MMPs) are a family of zinc-dependent endopeptidases responsible for tissue remodeling and the degradation of extracellular matrix proteins (30). In the vascular system, MMPs have the potential to degrade all major components of the vessel wall. MMP9 is secreted by various cells, including vascular smooth muscle cells, macrophages, and endothelial cells (31). Under the influence of inflammatory factors such as IL-17, IL-18, and oxidative stress, MMP9 is secreted in large amounts, initially in an inactive proenzyme form, which can be activated by various matrix metalloproteinases and proteases (31). The collagen, elastin, and other extracellular matrix proteins covering the necrotic core of plaques and the adjacent shoulder regions are degraded by MMP9, which leads to the weakening of the fibrous cap, accelerating plaque rupture and the subsequent clinical events, such as myocardial infarction and stroke (31). MMP9 not only promotes the infiltration of inflammatory cells due to ECM protein degradation but also facilitates the contact between vascular smooth muscle cells (VSMCs) and the matrix by degrading the ECM surrounding VSMCs. This leads to the transformation of VSMCs from a quiescent, contractile state into migratory and proliferative cells, which then form foam cells and further exacerbate atherosclerosis (31). Elevated MMP9 levels have been identified as an independent predictor of plaque vulnerability in coronary atherosclerotic heart disease, positively correlating with lipid necrotic core size and stenosis degree, with higher serum MMP9 levels observed in patients with plaque rupture (32, 33). Additionally, MMP9 is associated with the vulnerability of carotid atherosclerotic plaques (34). Previous studies have confirmed the correlation between MMP9 and plaque vulnerability in both the carotid and coronary arteries. This study shifts its focus to investigate the relationship between MMP9 and intracranial atherosclerotic plaque vulnerability, using imaging techniques to explore this association.

This study found that MMP9 levels were significantly higher in sICAS patients compared to healthy controls, and elevated MMP9 levels were associated with plaque enhancement, severe stenosis, and positive remodeling observed in high-resolution vessel wall imaging. The possible mechanism underlying the association between MMP9 and plaque enhancement, as well as severe stenosis, is the degradation of the extracellular matrix by MMP9, which promotes the migration of atherosclerotic and inflammatory cells to the lesion site, exacerbating the progression of atherosclerosis and the inflammatory response. In MMP9-silenced mouse models, serum MMP9 and high-sensitivity C-reactive protein (hs-CRP) levels were significantly reduced, suggesting that MMP9 deficiency may stabilize plaques by inhibiting inflammation (31). Studies on human carotid endarterectomy atherosclerotic plaques found that vascular endothelial growth factor (VEGF) and MMP9 were co-overexpressed in inflammatory cells (33). Research on porcine carotid atherosclerotic plaques found larger positive staining regions for MMP9 in plaques with significant neovascularization (35). These findings suggest that MMP9 may indirectly influence plaque stability by affecting neovascularization. Animal experiments also demonstrate the association between MMP9 and atherosclerosis. In the MMP9 transgenic rabbit model, increased infiltration of monocytes/macrophages and more prominent lipid core formation were observed in the aorta and coronary arteries (36). Histopathological analysis revealed that MMP9 is primarily located in the shoulder, necrotic core, and fibrous cap of atherosclerotic plaques, with higher levels and activity in unstable plaques compared to stable plaques (37).

In this study, MMP9 levels were significantly elevated in the positive remodeling group. This may be due to the role of MMP9 in arterial remodeling, as arterial remodeling requires the release of the structural scaffold imposed by the extracellular matrix (ECM). Furthermore, studies have shown that MMP9 staining is significantly higher in the positive remodeling segments of human coronary atherosclerotic plaques compared to negative remodeling segments (38). Aneurysmal arterial dilation may represent an extreme form of positive remodeling, with increased MMP9 expression detected in human thoracic aortic aneurysms (39). Additionally, Funayama et al. demonstrated that MMP9 levels negatively correlate with coronary artery fibrous cap thickness and positively correlate with the remodeling index (38).

NINJ1 and MMP9 are closely associated with the occurrence and progression of atherosclerosis. From a clinical perspective, NINJ1 and MMP9 hold promise as potential serum biomarkers for risk stratification in patients with intracranial atherosclerosis. Elevated serum levels of NINJ1 and MMP9 may help identify individuals at higher risk for vulnerable plaques, thereby assisting in the selection of patients who require further imaging evaluation or more aggressive preventive strategies. In this study, we determined the optimal cutoff values for NINJ1 and MMP9 through ROC curve analysis, providing preliminary evidence for their clinical applicability. However, further validation in larger prospective cohorts is needed to confirm their predictive value and explore how to effectively integrate these biomarkers into routine clinical workflows for personalized risk management.

In this study, although the combined AUC of NINJ1 and MMP9 in predicting plaque enhancement was statistically significant (AUC = 0.740), their discriminative ability remains moderate. This suggests that NINJ1 and MMP9 hold some value in reflecting intracranial plaque vulnerability, but their clinical application as standalone serum biomarkers may be limited. Studies have shown that hs-CRP levels are closely associated with the presence and severity of carotid and coronary atherosclerosis (40, 41). However, as a marker of systemic inflammation, hs-CRP still lacks sufficient specificity in evaluating localized vascular lesions. In contrast, NINJ1 and MMP9 are involved in the local inflammatory activation and structural disruption of atherosclerotic plaques and may serve as valuable complements to systemic inflammatory markers such as hs-CRP. Compared to advanced imaging techniques such as high-resolution vessel wall imaging (HR-VWI), which directly visualize plaque enhancement and remodeling features, serum biomarkers provide indirect assessments and may thus have lower discriminative accuracy. As standalone biomarkers, the clinical utility of NINJ1 and MMP9 may be limited; however, they hold promise as complementary tools in multimodal risk stratification strategies. Therefore, NINJ1 and MMP9 can be viewed as potential adjunct biomarkers to existing imaging techniques, and further prospective studies are needed to validate their predictive value and optimize their integration with imaging technologies for comprehensive plaque vulnerability assessment. Moreover, the evaluation of plaque vulnerability should also include lipid core size, fibrous cap thickness, and calcification. Unfortunately, our current imaging methods cannot quantify these features, but future studies could incorporate additional plaque vulnerability indicators.

## 5 Conclusion

NINJ1 and MMP9 are independent predictors of plaque enhancement, severe stenosis and positive remodeling in sICAS patients, with their combined predictive value being even higher. Both NINJ1 and MMP9 have the potential to serve as serum biomarkers for the vulnerability of intracranial atherosclerotic plaques.

## Data Availability

The raw data supporting the conclusions of this article will be made available by the authors, without undue reservation.
